# Placode and neural crest origins of congenital deafness in mouse models of Waardenburg-Shah syndrome

**DOI:** 10.1016/j.isci.2025.112400

**Published:** 2025-04-11

**Authors:** Jaime Tan, Alicia Duron, Henry M. Sucov, Takako Makita

## Main text

(iScience *28*, 111680; January 17, 2025)

In the initial publication of the article, an error occurred during the preparation and assembly of Figure 1C. Specifically, the y axis unit was DPOAE (dB SPL) but should be DPOAE (dBV). This now has been corrected and has been applied to both the online version and the PDF of the article. The authors have verified these changes and confirm that they do not affect the scientific conclusions of the study. The authors apologize for any confusion and inconvenience caused to readers.

**Figure:**
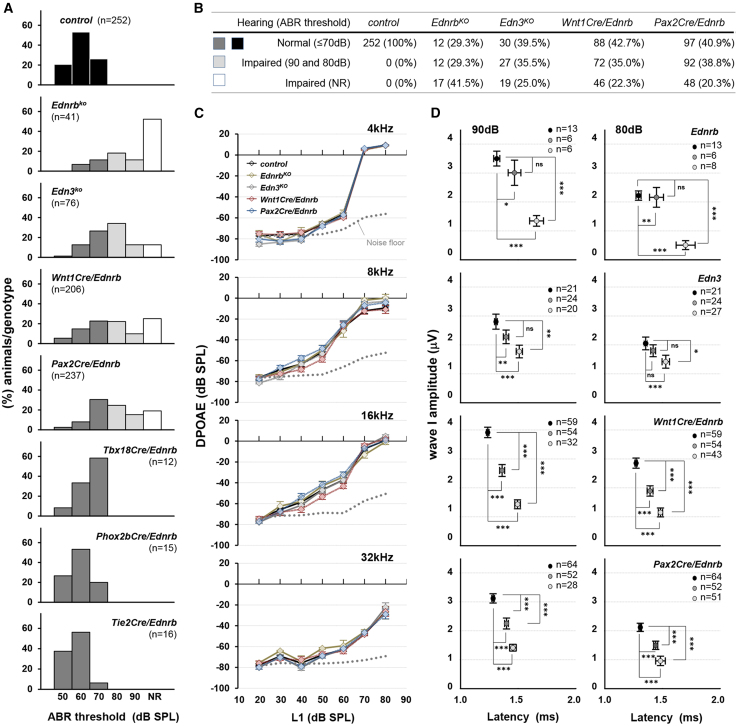
Figure 1. Incomplete penetrance of hearing impairment in Edn3-Ednrb signaling deficient mice (original)

**Figure:**
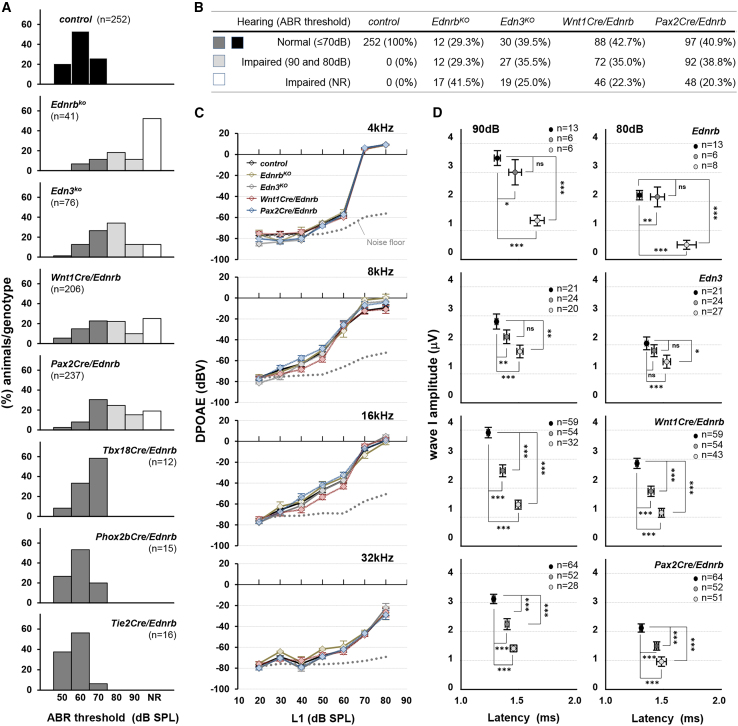
Figure 1. Incomplete penetrance of hearing impairment in Edn3-Ednrb signaling deficient mice (corrected)

