# Supplementary material for: Placode and neural crest origins of congenital deafness in mouse models of Waardenburg-Shah syndrome
Source: iScience. 2024 Dec 24;28(1):111680. doi: 10.1016/j.isci.2024.111680 (PMC11762213; doi:10.1016/j.isci.2024.111680)
Supplement: Document S1. Figures S1–S8 and Table S1 [file mmc1.pdf]

iScience, Volume *28*

Supplemental information

Placode and neural crest origins of congenital deafness in mouse models of Waardenburg-Shah syndrome

Jaime Tan, Alicia Duron, Henry M. Sucov, and Takako Makita

Genotype	n
Wild-type	37
Ednrb$^{+/-}$	8
Edn3$^{+/-}$	10
Wnt1Cre/Ednrb$^{fl/+}$	77
Pac2Cre/Ednrb$^{fl/+}$	88
Tbx18Cre/Ednrb$^{fl/+}$	9
Phox2bCre/Ednrb$^{fl/+}$	12
Tie2Cre/Ednrb$^{fl/+}$	11
Total	252

Table S1. A genotype breakdown of control mice used for ABR study. The control group in Figure 1 is composed of animals of these specific genotypes. The 37 wild-type mice include 16 littermates of *Ednrb* mutants and 21 littermates of *Edn3* mutants.

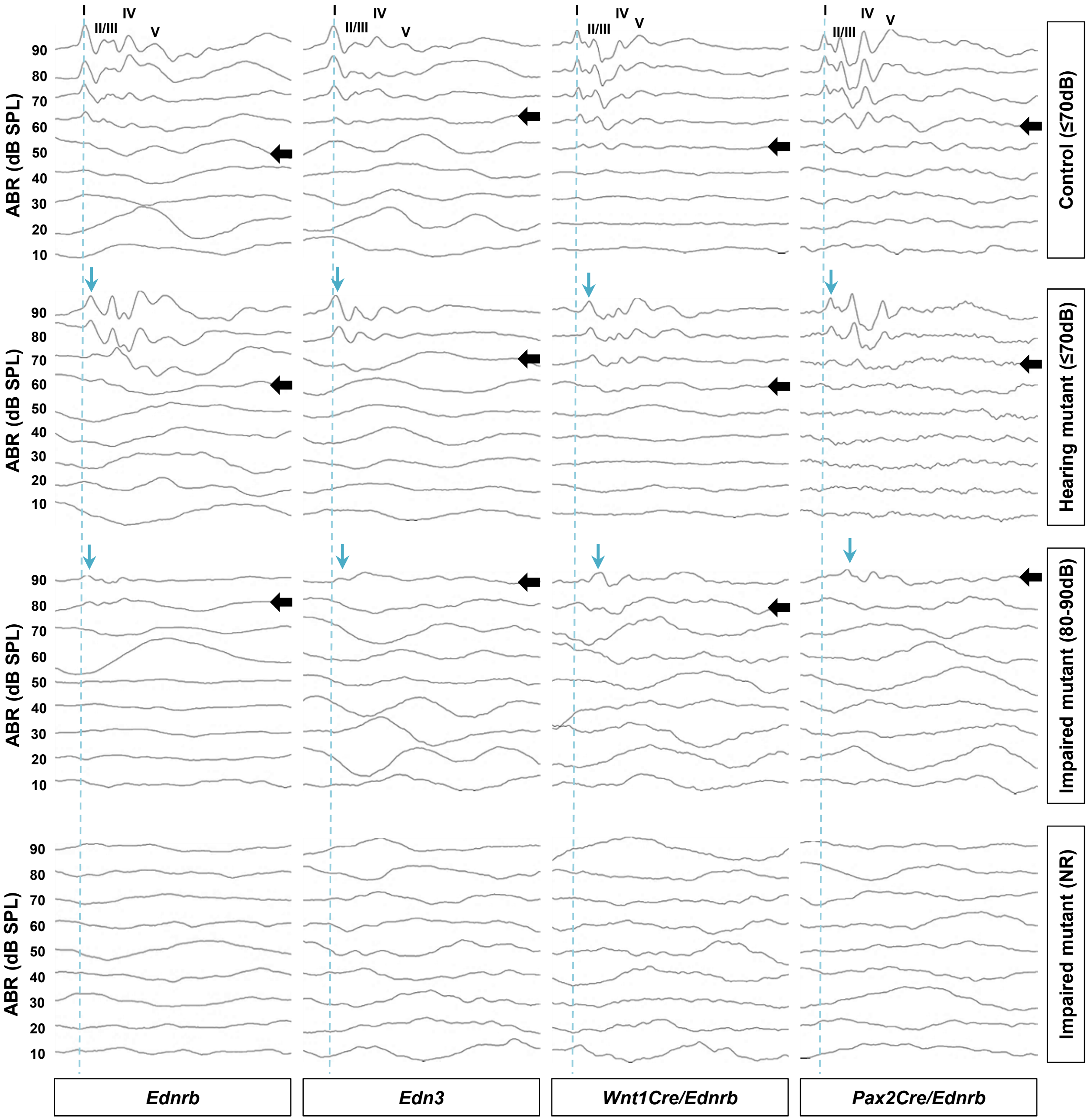

Figure S1. Representative ABR waveforms of P19 Edn3-Ednrb signaling mutants. Click-evoked ABR waveforms of individual animals. Because mutant mice were generated from different crosses, control mice and mutant mice with auditory phenotypes shown at right were from the same cohorts (shown at bottom) but not necessary from the same litters. The waveforms represent the responses in decreasing stimulus levels between 90dB and 10dB. Top row are the ABR waveforms of controls (see Table S1) from the indicated genetic backgrounds (i.e., littermates of mutant mice). Bottom three rows include example ABR waveforms of individual mutants from the indicated genetic background exhibiting normal hearing (ABR threshold≤70dB), impaired hearing (ABR threshold 80dB and 90dB), and total deafness (NR=no response) as indicated at the right. I-V denote the location of ABR peaks. The ABR threshold was determined as the lowest intensity at which a recognizable wave I waveform can be identified (individual threshold evaluation is noted by black arrows). Non-hearing (ABR=NR) was defined by the absence of auditory nerve response (wave I) at 90dB. Wave I represents spiral ganglion neuron activities. Blue dotted line indicates a time delay between stimulus input and SGN response in littermate control (latency). Blue arrows denote delays in wave I latency that were observed in hearing (ABR threshold≤70dB) and hearing impaired (ABR threshold 80-90dB) mutants. Compiled representations of wave I amplitude vs latency plots comparing controls vs mutants in each genetic background are shown in Fig. 1d.

a

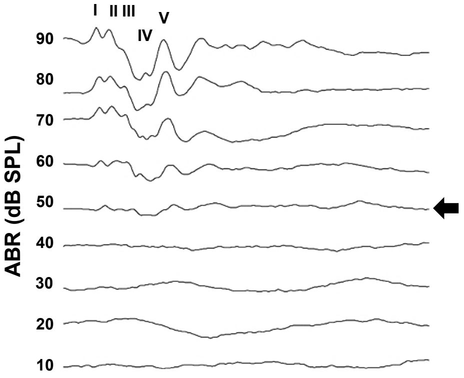

b

ABR Threshold	P18-19	P21-22
90	0	0
80	0	0
70	62 (24.6%)	0
60	135 (53.6%)	8 (19.5%)
50	54 (21.4%)	24 (58.5%)
40	1 (0.4%)	9 (22.0%)
30	0	0
20	0	0
10	0	0
Total number of animals	252	41

Figure S2. Developmental transition of hearing sensitivity in P18-19 vs. P21-22 control mice. (**a**) Click-evoked ABR waveforms in decreasing stimulus levels between 90dB and 10dB obtained from a P22 control mouse of ICR strain background. Black arrow point to the ABR threshold as 50dB. (**b**) The distribution of ABR threshold in P18-19 (n=252) vs. P21-22 (n=41) control mice. P18-19 control mice represents data sets compiled in Figure 1.

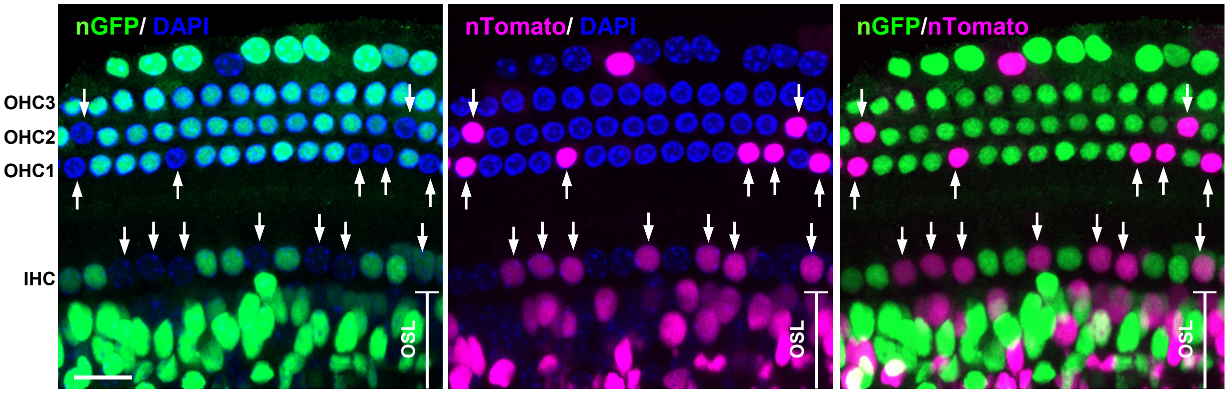

Figure S3. Recombination profile of *Pax2Cre* in cochlear hair cells. Wholemount cochlea preparation from P7 *Pax2Cre/R26*$^{nT\text{-}nG}$ mouse stained for GFP (green) and counterstained for DAPI (blue). Arrows point to the nuclei of non-recombined cells that express nuclear-Tomato (magenta). The osseous spiral lamina (OSL) area contains *Pax2Cre*-derived otic fibrocytes and auditory nerve associated Schwann cells which originate from non-*Pax2Cre* (*Wnt1Cre*) lineage (Fig. 3c). Scale bar; 20μm.

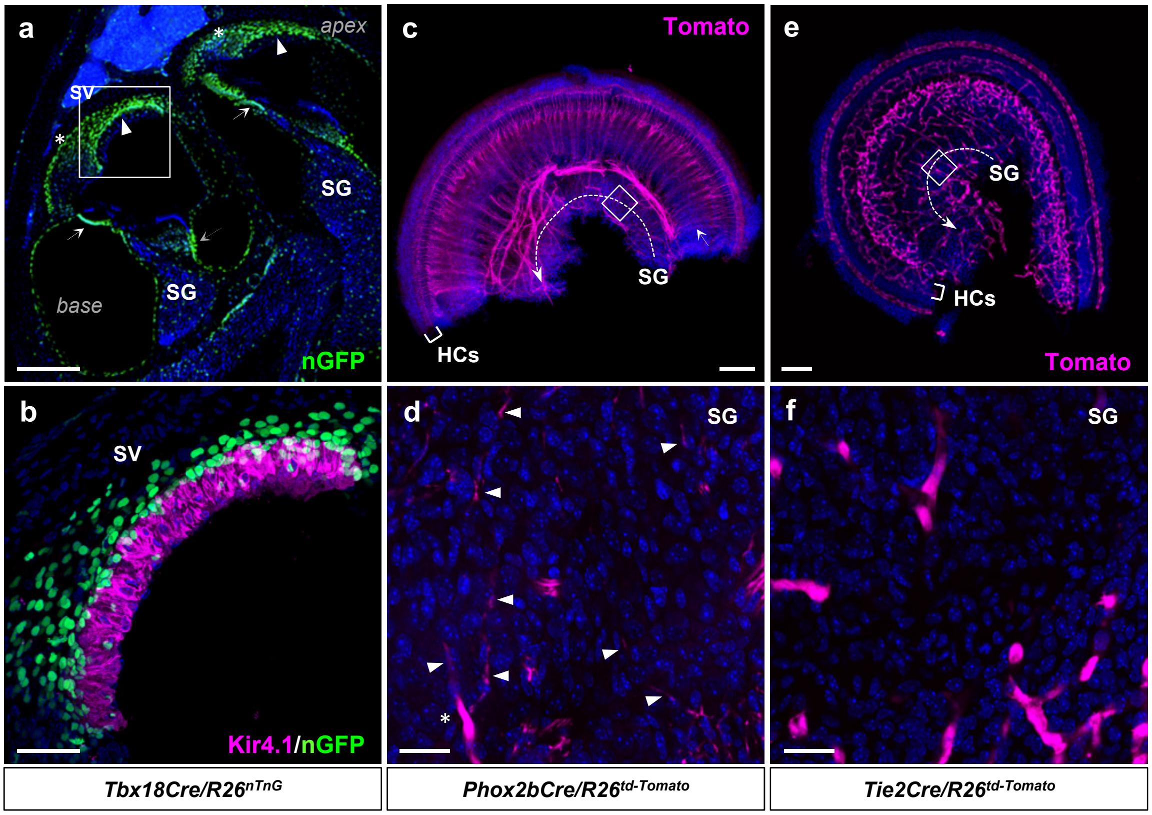

Figure S4. Recombination profile of *Tbx18Cre*, *Phox2bCre*, and *Tie2Cre* drivers. (**a**-**b**) Sagittal section of cochlea isolated from P19 *Tbx18Cre/R26*$^{nT\text{-}nG}$ mouse stained for GFP (green) and co-stained for Kir4.1 (magenta). (a) Low magnification view demonstrates *Tbx18Cre* activity in the stria vascularis (arrowheads) and otic mesenchyme derivatives including the basilar membrane (arrows), the lateral wall (asterisk) and the medial spiral limbus (dotted arrow). (b) High magnification of the boxed area in (a). *Tbx18Cre* labels fibrocytes in the spiral ligament and the basal stria cells which are located lateral to the Kir4.1^{+} intermediate stria cells. (**c**-**d**) Whole-mount preparation of cochlea isolated from a P0 *Phox2bCre/R26*$^{td\text{-}Tomato}$ mouse. Dotted arrow denotes the spiral ganglion (SG) and arrows point to the nerve fibers projecting towards hair cells (HCs). (d) High magnification view of the boxed area in (c). Tomato expression was not detected in neurons nor glia of the spiral ganglion, but observed in efferent nerve fibers (arrowheads) and an associated Schwann cell (asterisk) of the olivocochlear system. (**e**-**f**) Whole-mount preparation of cochlea isolated from a P0 *Tie2Cre/R26*$^{td\text{-}Tomato}$ mouse. Tomato signals represent endothelial cells of the cochlear vasculature. (f) High magnification view of the bracketed area in (e). Scale bars: 200µm (a), 100µm (b, c, e), 20µm (d, f).

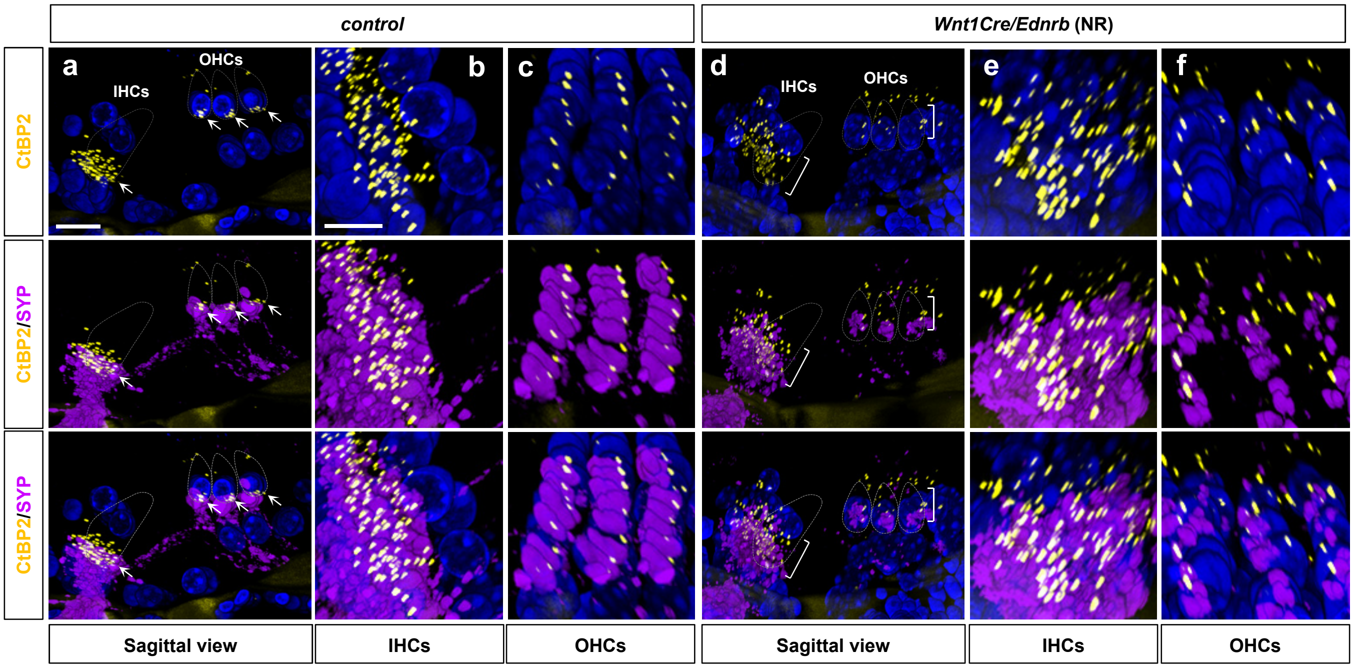

Figure S5. Defective pre- and post- synaptic assembly in hearing impaired *Wnt1Cre/Ednrb* mutant mice. (**a**, **d**) Confocal z-stack images from 100 μm sagittal sections of cochlea isolated from P19 deaf *Wnt1Cre/Ednrb* mutant (d) and its littermate control (a) stained for CtBP2 (yellow), co-stained for SYP (magenta) and counterstained for DAPI (blue). Dotted lines denote outlines of IHC and OHCs of the first row. (a) Arrows point to the condensed localization of CtBP2-containing synaptic ribbons alongside the SYP^{+} afferent nerve endings at the basolateral side of the control hair cells. High magnification of the rotated views of IHC and OHC synapses in (a) are shown in (b) and (c), respectively. A 100 μm section contains 8 rows of IHCs (b) and OHCs (c), and their respective afferent synapses. (d) Brackets denote scattered and disorganized localization of CtBP2 puncta within the IHC and OHCs of the first row in the *Wnt1Cre/Ednrb* mutant cochlea. High magnification of the rotated views of basolateral surface of the IHC and OHCs in (d) are shown in (e) and (f), respectively. Scale bars: 20μm (a, d), 10μm (b, c, e, f).

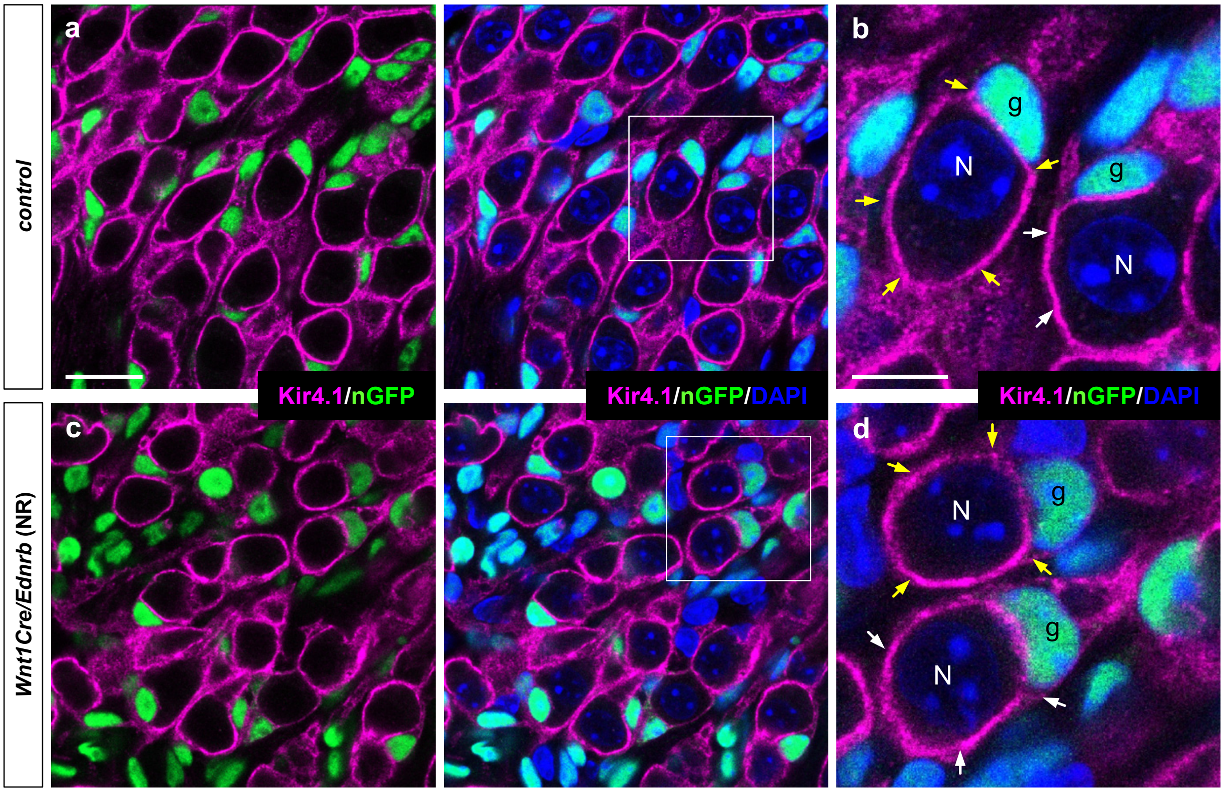

Figure S6. No obvious disruption in distribution and morphology of *Ednrb*-deficient satellite cells in the spiral ganglia of hearing impaired *Wnt1Cre/Ednrb* mutant mice. Confocal images of spiral ganglia isolated from P19 *Wnt1Cre/Ednrb/R26*$^{nT\text{-}nG}$ mutant (c; ABR=NR) and a littermate control (a) stained for GFP (green), co-stained for Kir4.1 (magenta), and counterstained for DAPI (blue). High magnification views of bracketed areas in (a) and (c) are shown in (b) and (d), respectively. "N" and "g" in (b) and (d) denote two individual spiral ganglion neurons and their associated satellite glia, respectively. Yellow and white arrows in (b) and (d) point to Kir4.1$^+$ glial processes that ensheath their associated neurons. Scale bars: 20μm (a, c), 10μm (b, d).

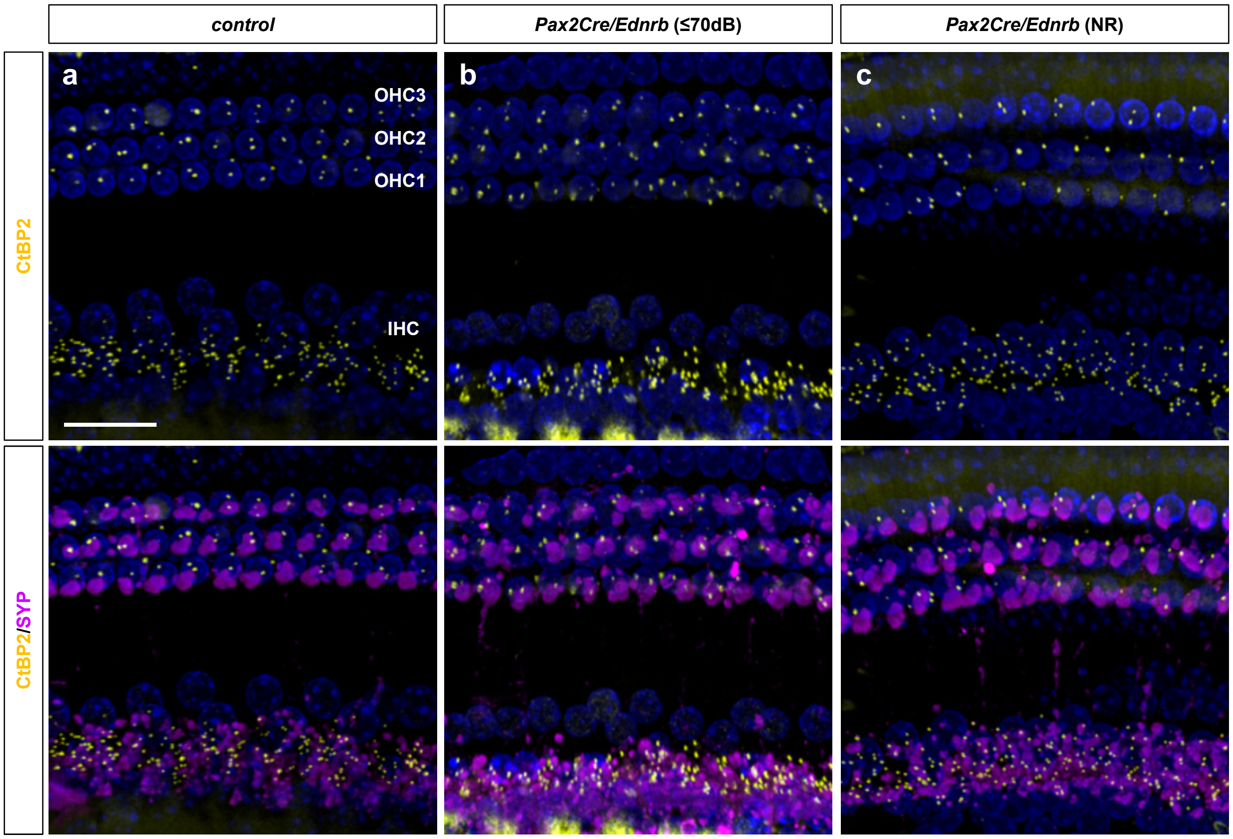

Figure S7. Normal pre- and postsynaptic organization in hearing impaired *Pax2Cre/Ednrb* mutant mice. (**a-c**) Whole-mount preparation of cochlea isolated from P19 hearing *Pax2Cre/Ednrb* (b), hearing impaired *Pax2Cre/Ednrb* (c) mutant mice and a littermate control (a) immunostained for CtBP2 (yellow), SYP (magenta) and DAPI (blue). Scale bar: 50µm (a-c).

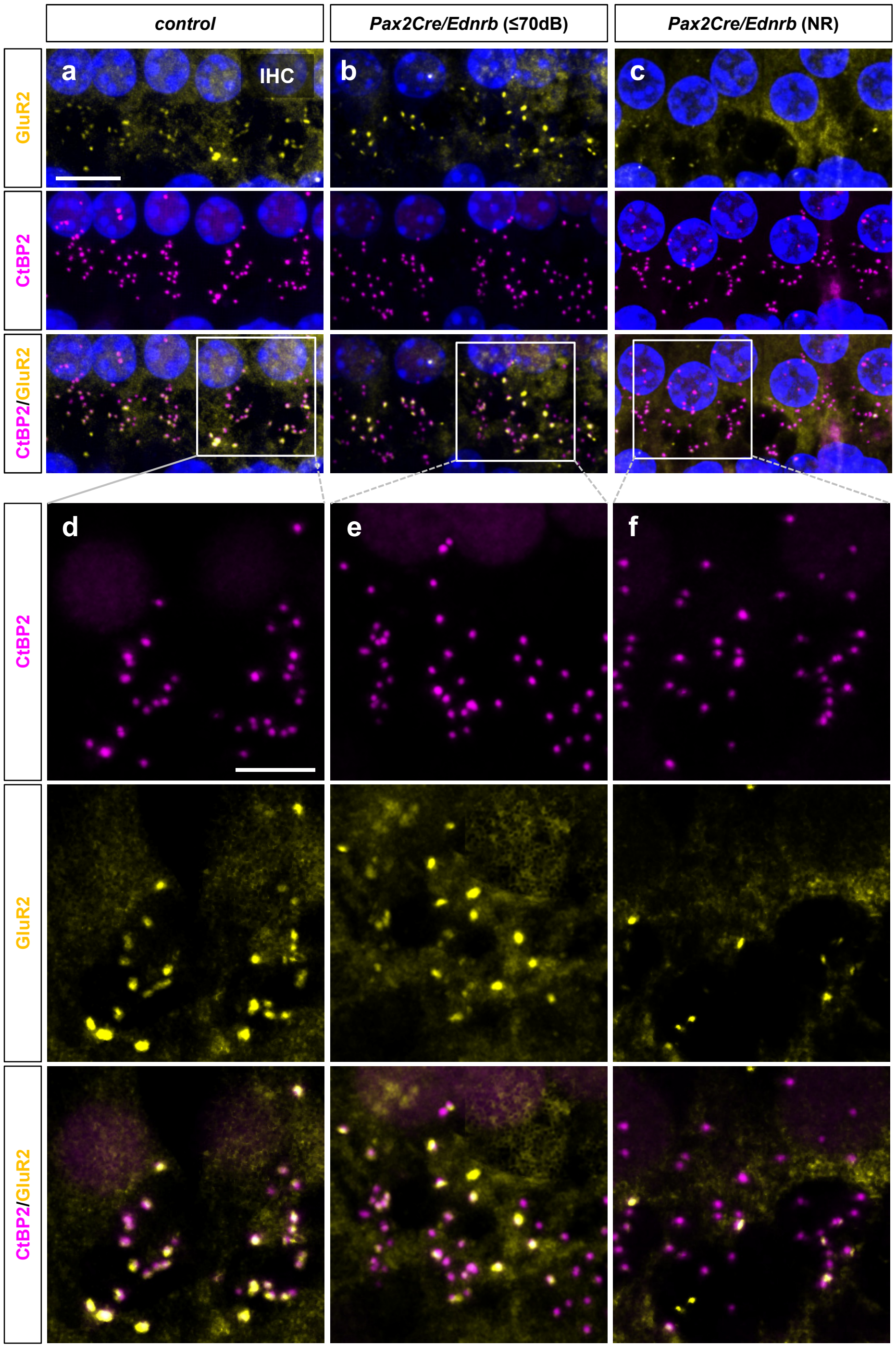

Figure S8. Reduced postsynaptic GluR2 in hearing-impaired *Pax2Cre/Ednrb* mutant IHC. (**a-c**) Whole-mount preparation of cochlea isolated from P19 hearing *Pax2Cre/Ednrb* (b), hearing impaired *Pax2Cre/Ednrb* (c) mutants and a littermate control (a) immunostained for GluR2 (yellow), and CtBP2 (magenta), and counterstained with DAPI (blue). Magnified views of bracketed area in a-c are shown in **d-f**. Scale bars: 10μm (a-c), 5μm (d-f).
